# Integrated Identification of Macrophage NF-κB, p38 MAPK and AKT1 Signalling as Candidate Targets of Zerumbone in Experimental Periodontitis

**DOI:** 10.3390/cimb48100985

**Published:** 2026-09-24

**Authors:** Ting Long, Fang Dai, Xiaoxue Wang, Yichen Hu, Meixiu Jiang, Guoping Cheng, Kaiqiang Yang, Li Li, Zixuan Wang, Yaowen Huang, Li Song

**Affiliations:** 1 Center of Stomatology, The Second Affiliated Hospital, Jiangxi Medical College, Nanchang University, Nanchang 330006, China; longting920318@163.com (T.L.); ndefy11338@ncu.edu.cn (F.D.); 17326915979@163.com (X.W.); ndefy24141@ncu.edu.cn (Y.H.); chengguoping2022@outlook.com (G.C.); yangkaiqianghappy@163.com (K.Y.); 403007220047@email.ncu.edu.cn (L.L.); zixuandongying@163.com (Z.W.); hyaowen1@163.com (Y.H.); 2 Jiangxi Provincial Key Laboratory of Oral Diseases, The Second Affiliated Hospital, Jiangxi Medical College, Nanchang University, Nanchang 330006, China; 3 JXHC Key Laboratory of Periodontology, The Second Affiliated Hospital of Nanchang University, Nanchang 330006, China; 4 The Institute of Periodontal Disease, Nanchang University, Nanchang 330006, China; 5 The Second Clinical Medical School, Nanchang University, Nanchang 330006, China; 6 The National Engineering Research Center for Bioengineering Drugs and the Technologies, Institute of Translational Medicine, Jiangxi Medical College, Nanchang University, 999 Xuefu Road, Nanchang 330031, China; jiangmxs@ncu.edu.cn

**Keywords:** periodontitis, zerumbone, network pharmacology, macrophage polarisation, immunomodulation

## Abstract

Characterised by progressive alveolar bone loss, periodontitis arises from immune dysregulation and is classified as a chronic inflammatory condition. Zerumbone (Zer) has anti-inflammatory properties; however, its mechanism within the periodontal context remains unclear. We investigated the therapeutic effects and potential molecular targets of Zer by integrating *in vivo* efficacy assessment (using male C57BL/6 mice), network pharmacology, molecular docking, and molecular dynamics simulations, together with analysis of public single-cell transcriptomic data, with mechanistic validation using RAW 264.7 macrophages stimulated with LPS. In vivo, Zer attenuated bone resorption, preserved collagen integrity, reduced the number of TRAP-positive osteoclasts, and mitigated inflammation without hepatorenal toxicity. Network pharmacology predicted *NFKB1*, *MAPK14*, and *AKT1* as potential core targets, while IL-17 pathway enrichment suggested the involvement of *IL17A*; single-cell data localised *NFKB1/MAPK14/AKT1* to macrophages and showed that *IL17A* is predominantly expressed in T cells. In vitro, Zer dose-dependently suppressed LPS-driven phosphorylation of NF-κB p65, p38 MAPK and AKT1, correlating with reduced pro-inflammatory cytokine release and an M1-to-M2 phenotypic shift. In vivo, Zer also reduced gingival IL-17A expression. However, as IL-17A is primarily produced by T cells and our in vitro experiments were conducted exclusively in macrophages, this observation remains correlative and does not establish a direct T-cell-mediated effect. Collectively, these findings suggest that Zer ameliorates periodontitis partly through suppression of macrophage NF-κB and p38 MAPK signalling, with reduced AKT1 phosphorylation observed alongside these effects; however, whether AKT1 causally contributes to these effects remains to be determined. In addition, the potential impact on T-cell-associated IL-17A responses warrants further investigation.

## 1. Introduction

Periodontitis, a chronic inflammatory disorder, progressively destroys the periodontal tissues. In 2021, severe periodontitis had an age-standardised prevalence of roughly 12–13% worldwide [1]. It is associated with systemic disorders such as atherosclerosis, diabetes mellitus, rheumatoid arthritis and Alzheimer’s disease [2,3], and places a substantial burden on patients and health care systems. Its pathogenesis is multifactorial, involving bacterial infection, host immune dysregulation, and genetic and environmental influences [4]. Among these, aberrant immune activity—particularly involving macrophages and T cells—is central to driving local inflammation and alveolar bone resorption [5,6]. Macrophages release pro-inflammatory cytokines that sustain tissue destruction [7], while T cells contribute through IL-17 production, which promotes osteoclastogenesis and bone loss [5]. These processes are regulated by intracellular signalling cascades, including PI3K/AKT, NF-κB and MAPK pathways [8].

Contemporary clinical practice relies heavily on mechanical debridement. Root planing and scaling constitute the mainstay of periodontal treatment [9], but mechanical means alone cannot fully eliminate pathogens, as certain pathogens can invade soft tissues or colonise other oral surfaces [10,11]. Residual bacterial burden can maintain innate immune activation and low-grade chronic inflammation, and it is this inflammation—rather than the bacteria themselves—that drives much of the tissue destruction [12]. Adjunctive pharmacotherapy is therefore often required, but current options rely mainly on antibiotics, which carry risks of antimicrobial resistance and disturbances to the oral microbiome [13,14]. Safer and more effective host-modulatory agents—particularly those capable of suppressing both dysregulated inflammation and bone resorption—are therefore needed [8,15].

Zerumbone (Zer) is a natural compound from Zingiberaceae plants, a family traditionally used for inflammatory conditions [16]. It has anti-inflammatory and tissue-regenerative properties [17,18]: in cell models, it reduces IL-1β and inhibits NLRP3 inflammasome activation [19]; it also promotes corneal wound repair by downregulating STAT3 and MCP-1 [18], and a Zer-containing gel has shown efficacy in diabetic rat models [20]. Network pharmacology has been applied to natural products in periodontitis, including shikonin and *Coptidis rhizome* [21,22], and has also predicted Zer’s interactions with TGF-β1 and GAD67/gephyrin in other disease models [23,24]. However, no study has yet investigated Zer in periodontitis using network pharmacology combined with experimental validation, nor have the predicted targets been experimentally assessed. In particular, whether Zer acts on specific cell types within the periodontal microenvironment remains unexplored.

Here we assessed the in vivo efficacy of Zer in a male mouse model of periodontitis and examined its effects on macrophage signalling in vitro, alongside computational target prediction and single-cell transcriptomic localisation. Our findings may inform the development of Zer as an adjunctive immunomodulatory therapy for periodontitis.

## 2. Materials and Methods

### 2.1. Chemicals and Reagents

Zer, a known sesquiterpenoid, was purchased from Dexter Biotech (Chengdu, China) (lot number DST200630-145, high-performance liquid chromatography (HPLC) indicated a purity of >98%; see COA in Supplementary Materials File S1). The stock solution (3 mM) was generated via dissolution with dimethyl sulfoxide (DMSO) and kept at −20 °C. For cell-based assays, working concentrations (3, 15, and 30 μM) were prepared by mixing the stock solution with culture medium. Zer was prepared as a solution in corn oil (C14030; Aladdin Reagents, Shanghai, China) for the in vivo experiments.

### 2.2. Porphyromonas gingivalis (Pg) Culture

*Pg* W83 was cultivated as described previously [25], with brief details as follows. Bacterial cultures were maintained in brain-heart infusion broth (028360, HuanKai Microbial, Guangzhou, China) supplemented with vitamin K3 (A502486-0100, Sangon Biotech, Shanghai, China) and hemin (A602521-0025, Sangon Biotech, Shanghai, China). The culture medium (pH ~7.0) was kept in an anaerobic chamber (37 °C) under an atmosphere of 5% H_2_, 5% CO_2_, and 90% N_2_ for *Pg* cultivation. Once a reading of optical density (OD) at 600 nm was obtained as 0.5 (at the log growth phase), the bacterial culture was subjected to centrifugation (4000× *g*, 10 min), after which the pellet was resuspended in 100 μL of 2% sodium carboxymethyl cellulose (CMC) at approximately 10^9^ colony forming units (CFU)·mL^−1^.

### 2.3. Animals, Periodontitis Model Induction and Drug Treatment

The procedures involving experimental animals were reviewed and granted ethical approval by the Ethics Committee of Nanchang University (approval number NCULAE-20221031158, approved on 31 October 2022). This study is part of a larger project covered by the same ethics approval, and the findings reported here are derived from that single approval. All animal procedures followed the standard operating procedures and animal care policies mandated by Nanchang University, and were reported following the ARRIVE guidelines 2.0. 

Male C57BL/6 mice aged six to eight weeks obtained from Collective PharmaCare Laboratory Animal Technologies (Nanjing, China) were maintained in a specific pathogen-free animal facility. In this study, we used only male mice, aiming to reduce variability associated with the oestrous cycle, although this restricts the generalisability of our findings to female mice. The animal facility was maintained under conditions of 22 ± 2 °C, 50 ± 10% relative humidity, and a 12-h photoperiod (light/dark). After one week of acclimatisation, the animals were assigned randomly to three groups (*n* = 8 per group, based on the standard range used in murine periodontitis studies and the 3Rs principle) by means of a computer-generated random number table: the control (Con), periodontitis (CP), and periodontitis + Zer (CP+Zer) groups. The Con group was locally administered 100 µL of 2% CMC as a sterile carrier. In the CP group, periodontitis was induced as follows: under intraperitoneal anaesthesia with tribromoethanol (Avertin; Dowobio, Shanghai, China), the bilateral maxillary second molars underwent ligation using 5-0 silk sutures placed around them. The surrounding gingival sulci were then locally inoculated with 100 µL of 2% CMC containing freshly resuspended *Pg* (~1 × 10^9^ CFU·mL^−1^) three times per week for four weeks. After the 4-week induction period, the silk sutures were carefully removed. Zer (20 mg·kg^−1^) was then dissolved in corn oil by ultrasonication in a 25 °C water bath for 10 min, yielding a pale-yellow solution. This dose was selected based on prior studies in male mice (C57BL/6J and ICR) demonstrating its efficacy and safety [23,26]. This solution was given once daily by oral gavage to the CP+Zer group, and was vortexed thoroughly before each use to ensure uniform mixing. The Con and CP groups received an equal volume of corn oil alone. The gavage operator was blinded, as the Zer solution and vehicle were visually indistinguishable.

Upon reaching the predetermined two-week endpoint, male mice were deeply anaesthetised by inhalation of isoflurane. Briefly, animals were placed in a sealed induction chamber containing isoflurane-soaked cotton wool. Anaesthesia was maintained until loss of the pedal withdrawal reflex (response to toe pinch) and establishment of a regular respiratory pattern, confirming a surgical plane of anaesthesia prior to all procedures. Under this deep anaesthesia, blood was collected via orbital sinus exsanguination, and every effort was made to minimise suffering. Following exsanguination, cervical dislocation was performed to ensure death, and tissue samples were subsequently harvested. These samples were coded prior to analysis. The final number of samples included in each analysis varied because some samples were excluded due to technical quality issues (e.g., poor histological section quality, insufficient RNA yield, serum haemolysis, or insufficient sample volume). The exact n for each analysis is reported in the corresponding figure legend. All outcome assessments and statistical analyses were performed by investigators blinded to group allocation.

### 2.4. Micro-Computed Tomography (Micro-CT) Assessment of Alveolar Bone

Following euthanasia, the maxillae underwent fixation in 4% paraformaldehyde and were subsequently imaged using a micro-CT device (VivaCT40, SCANCO Medical AG, Brüttisellen, Switzerland) at a 12.5 μm resolution. Three-dimensional (3D) visualisation of the CT images was performed with Mimics software (Materialise, version 21.0). Bone loss in the alveolar ridge was assessed by quantifying the average distance from the cementoenamel junction (CEJ) to the alveolar bone crest (ABC) at six predefined points around the upper second molar: mesiobuccal, mid-buccal, distobuccal, mesiopalatal, mid-palatal and distopalatal. For each specimen, the BV/TV ratio (bone volume divided by tissue volume) was also computed.

### 2.5. Histological and Immunohistochemical (IHC) Analyses

Following micro-CT imaging, the maxillary specimens underwent decalcification for histological assessment, prior to paraffin embedding and sectioning. We performed Haematoxylin and Eosin (H&E) staining of tissue sections using a commercial H&E staining kit (G1005, Servicebio, Wuhan, China), adhering to the supplier’s recommended protocol. Osteoclast identification was performed through tartrate-resistant acid phosphatase (TRAP) staining with a commercial kit (G1050, Servicebio). Periodontal tissue was subjected to staining with a Masson’s trichrome staining kit (G1006, Servicebio). Images of H&E-, TRAP- and Masson’s trichrome-stained tissues were visualised and imaged under an optical microscope (Olympus, Tokyo, Japan). Osteoclasts positive for TRAP situated within the tissue sections between the first and second molars were independently quantified by two investigators. IL-17A expression within periodontal tissues was evaluated using IHC. Briefly, maxillary sections were treated with a rabbit anti-IL-17A monoclonal antibody (GB11110-1-100, Servicebio) at 4 °C overnight in a humidity-controlled chamber. After removal of unbound primary antibody by PBST washing, an incubation of the sections with HRP-labelled goat anti-rabbit secondary antibody (Servicebio) was performed at room temperature for 1 h, followed by staining with the DAB substrate. The integrated optical density (IOD) of IL-17A immunostaining was semi-quantitatively assessed using ImageJ (version 1.8.0) and presented as IOD (×10^4^).

### 2.6. Identification of Potential Zer Targets and Periodontitis-Related Genes

We identified potential target genes of Zer from the SwissTargetPrediction, GeneCards, and SEA databases. In parallel, potential target genes for periodontitis were retrieved from the GeneCards, OMIM, DisGeNET, and CTD databases. Corresponding URLs are listed in the Supplementary Materials File S2.

### 2.7. Hub Target Identification, Protein–Protein Interaction (PPI) Network Construction, and Pathway Enrichment

The initial targets of Zer for periodontitis treatment were determined using Venny 2.1. A PPI network diagram was generated through submission of the overlapping targets to the STRING database, with its organism specified as *Homo sapiens* [27]. TSV Files were acquired from the STRING database and then processed using Cytoscape 3.9.1 to screen key targets. Overlapping targets underwent Gene Ontology (GO) term enrichment alongside Kyoto Encyclopedia of Genes and Genomes (KEGG) pathway analyses, conducted with the R package “clusterProfiler” (version 3.16.1). Cytoscape 3.9.1 was employed to generate a Zer–target–pathway network, which links Zer, 72 overlapping targets, and ten KEGG pathways associated with the inflammatory response (adjusted *p* < 0.05). Corresponding URLs are listed in the Supplementary Materials File S2.

### 2.8. Single-Cell RNA Sequencing (scRNA-Seq) Analysis of Hub Genes in Periodontitis

The Seurat package (version 4.3.1) was utilised within the R computational framework to analyse publicly available scRNA-seq data, with particular focus on the expression profiles of four identified hub genes. The analysis utilised the GSE171213 dataset, sourced from the Gene Expression Omnibus (GEO) database [28]; expression quantification for the selected genes was performed across distinct cell populations in healthy control (HC) and periodontally diseased (PD) human tissues to identify the cell types with enriched expression, thereby guiding subsequent mechanistic investigations. The expression profiles of these genes across the different cell populations were visualised using dot plots. These dot plots represent the gene expression profiles across distinct cell populations in both HC and PD tissues.

### 2.9. Molecular Docking

Molecular docking was used to investigate the binding mechanism between the ligand and its target protein [29]. Briefly, the 3D structures of these following proteins were obtained from the RCSB Protein Data Bank (PDB) (corresponding URLs are provided in the Supplementary Materials File S2): NF-κB p50 (PDB ID: 3GUT), p38 MAPK (PDB ID: 1A9U), AKT1 (PDB ID: 4GV1), and IL-17A (PDB ID: 7WKX). Non-essential components, including water, ions, and co-crystallized ligands, were removed, and the structures were energy-minimised using AutoDock 4.2 software. Subsequently, molecular docking was conducted between the processed protein structures and the ligand using the same software. This analysis evaluated key binding parameters—including binding energy and molecular interactions—between the drug functional groups and receptor residues. Molecular visualisation of the docking results was subsequently carried out via PyMOL software (version 3.1.5.1).

### 2.10. Molecular Dynamics (MD) Simulation and Stability Analysis

GROMACS 2023.5 was employed to perform MD simulations to evaluate the dynamic stability of Zer bound to NF-κB p50, p38 MAPK, AKT1, and IL-17A. The receptor proteins were assigned the Amber99SB-ILDN force field, while the topology of Zer was generated with Sobtop based on the GAFF force field, with partial charges derived from the RESP fitting procedure. Each complex was solvated within a cubic box of TIP3P water (1.0 nm margin), and neutralisation was achieved with Na^+^ and Cl^−^ ions. Energy minimisation (50,000 steps, steepest descent) was followed by 100 ps NVT (V-rescale, 310.15 K, τ = 0.1 ps) and 100 ps NPT (Parrinello–Rahman, 1 bar, τ = 2.0 ps) equilibration, during which position restraints (with a force constant of 1000 kJ·mol^−1^·nm^−2^) were imposed on the protein’s heavy atoms. After releasing the restraints, a 100 ns production run was conducted under the same NPT conditions. Long-range electrostatics were handled via the PME approach (cut-off 1.0 nm, with dispersion correction). Trajectories were analysed for RMSD, RMSF, Rg, and SASA for assessing conformational stability, residue flexibility, structural compactness, and solvent accessibility.

### 2.11. Cell Culture

At 37 °C under 5% CO_2_ with standard growth conditions, RAW 264.7 cells (obtained from the Chinese Academy of Sciences Cell Bank) were maintained in DMEM with 10% FBS and 1% penicillin–streptomycin. Cells were subcultured by gently dislodging the adherent monolayer with fresh culture medium using a pipette. Cells at passage three were used for experiments.

### 2.12. Cell Viability Assay

Cell viability was quantified employing a CCK-8 kit (SB-CCK8, ShareBio, Shanghai, China). RAW 264.7 cells were exposed to Zer (0, 3, 15, or 30 μM) for 24 h. The cells were then treated with CCK-8 reagent (10 μL in 100 μL of fresh medium) for 1 h. The SpectraMax Plus microplate reader (Molecular Devices, San Jose, CA, USA) was employed to record absorbance at 450 nm. A group without cells was used as the blank control. Three independent experiments were performed, each with five parallel wells per group. Cell viability (%) was computed from the mean absorbance of five parallel wells per group (blank-corrected) as: (mean ODtreatment − mean ODblank)/(mean ODcontrol − mean ODblank) × 100, relative to the untreated control.

### 2.13. Quantitative Real-Time PCR (qRT-PCR)

The transcription of periodontitis-related genes in gingival tissues and M1/M2 polarisation-associated genes in RAW 264.7 cells were analysed via qRT-PCR. Cells were exposed to Zer (3 μM and 15 μM) along with 1 μg·mL^−1^ *Pg*-lipopolysaccharide (LPS) (tlrl-pglps, InvivoGen, Toulouse, France) for 24 h. Table 1 provides the primer sequences. qRT-PCR was performed as follows: total RNA was subjected to reverse transcription with the MonScript^TM^ RT All-in-One Mix with dsDNase (Monad Biotech, Wuhan, China). Quantification was performed with SYBR Green PCR Master Mix (Monad Biotech) on a QuantStudio real-time PCR system (Thermo Fisher Scientific, Waltham, MA, USA). Relative transcript levels were calculated via the 2^−ΔΔCt^ method, with Ct denoting the cycle threshold.

### 2.14. Enzyme-Linked Immunosorbent Assay (ELISA) and Serum Biochemistry

Blood samples were collected after euthanasia, and serum was isolated for quantification of IL-1β, IL-6, TNF-α and MPO using commercial ELISA kits (Enzyme Link Bio, Shanghai, China) with the following catalogue numbers: IL-1β (ML720174), IL-6 (ML063159), TNF-α (ML720852) and MPO (ML728122). The same serum samples were used for measurement of aspartate aminotransferase (AST), alanine aminotransferase (ALT), blood urea nitrogen (BUN) and creatinine (Cr) using an automated biochemical analyser (Rayto Life Sciences, Shenzhen, China) according to the manufacturer’s instructions. The same serum samples were used for measurement of alanine aminotransferase, aspartate aminotransferase, creatinine and blood urea nitrogen using an automated biochemical analyser according to the manufacturer’s instructions. For cell experiments, RAW 264.7 cells were stimulated with Zer (3 μM and 15 μM) together with *Pg*-LPS for 12 h. The culture supernatants were subsequently harvested, spun at 1000× *g* for 10 min, and analysed for IL-1β, TNF-α and IL-6 via ELISA. Cytokine concentrations were determined from the standard curve.

### 2.15. Cell Immunofluorescence (IF) Staining

RAW 264.7 cells were seeded onto glass coverslips and stimulated with Zer (3 μM and 15 μM) and *Pg*-LPS for 24 h, after which they were fixed for 10 min at 4 °C in 4% paraformaldehyde, permeabilised using 0.2% Triton X-100 (10 min), followed by blocking with 2% BSA for 2 h, with phosphate-buffered saline (PBS) washes between steps. Overnight incubation with primary antibodies was performed at 4 °C on coverslips placed in a humidified chamber. After a PBS wash on the next day, the cells were exposed to fluorescently labelled secondary antibodies for 2 h at room temperature, and nuclei were labelled with DAPI. After mounting onto slides, images were obtained with a fluorescence slide scanner (3DHISTECH, Budapest, Hungary). Mean fluorescence intensity was measured for each image and normalised to the control to obtain relative fluorescence intensity (fold of control).

### 2.16. Western Blot (WB) Analysis

RAW 264.7 cells were treated with Zer (3 μM and 15 μM) in the presence of 1 μg·mL^−1^ *Pg*-LPS for 30 or 60 min. Cell lysis was performed with RIPA buffer enriched with a protease inhibitor cocktail (both from Servicebio), and protein content was determined via a BCA assay (Yeasen, Shanghai, China). Equivalent protein aliquots were resolved by SDS-PAGE on 10% gels (Yeasen, Shanghai, China) and blotted onto PVDF membranes (Millipore, Billerica, MA, USA). Following blocking with rapid blocking buffer (ShareBio), the membranes were probed overnight at 4 °C with primary antibodies against GAPDH (10494-1-AP, 1:10,000, Proteintech, Wuhan, China), phospho-NF-κB p65 (310013, 1:1000, Zen-bio, Chengdu, China), NF-κB p65 (380172, 1:1000, Zen-bio), phospho-p38 MAPK (310091, 1:1000, Zen-bio), p38 MAPK (200782, 1:1000, Zen-bio), phospho-AKT1 (310021, 1:1000, Zen-bio), and AKT1 (342529, 1:1000, Zen-bio). Subsequently, the membranes were subjected to triple TBST washing and then incubated with the corresponding secondary antibody (RGAR001, 1:10,000, Proteintech) for 2 h. Following visualisation with an enhanced chemiluminescence kit (SQ201, Epizyme Biotech, Shanghai, China), quantification of the protein bands was carried out using ImageJ. For quantification, each band was first normalised to its corresponding GAPDH loading control, then expressed relative to the control group mean within the same experiment. All conditions were examined in three independent experiments.

### 2.17. Statistical Analysis

Data are presented as mean ± standard deviation and subsequently analysed with GraphPad Prism 9. For multiple group comparisons, one-way ANOVA with Tukey’s post hoc test was employed. To control for multiple testing across all key comparisons, the Benjamini–Hochberg false discovery rate (FDR) procedure was applied. Significance levels in the figures (i.e., asterisks) are based on uncorrected *p* values; comparisons that did not remain significant after FDR correction are shown as “ns”. All other comparisons that were significant before FDR correction remained significant after correction (*q* < 0.05). Each experiment was performed at least three times, with significance set at *p* < 0.05 (or *q* value < 0.05 for FDR-adjusted analyses).

## 3. Results

### 3.1. Zer Attenuated Alveolar Bone Resorption in Male Mice with Periodontitis

To evaluate the therapeutic potential of Zer, periodontitis was induced via ligature and topical *Pg* delivery. Two groups of male mice then received daily gavage of either Zer or corn oil (Figure 1A,B). Micro-CT assessment revealed that the CP group exhibited a significantly larger CEJ-ABC distance and a significantly lower BV/TV ratio compared with the Con group, confirming successful periodontitis induction (Figure 1C,D(a),D(b)). Crucially, oral administration of Zer significantly reduced the CEJ-ABC distance and restored BV/TV values relative to the CP group (Figure 1D(a),D(b)), indicating that Zer alleviates alveolar bone loss.

Masson staining revealed that the CP group showed a marked decrease in the density of blue-stained collagen fibres with disorganised arrangement, whereas after Zer treatment, the fibre density was relatively increased and the architecture appeared more organised (Figure 1E).

Osteoclast activity was further evaluated by TRAP staining. TRAP-positive osteoclasts were more abundant in the CP group compared with the Con group. Notably, the CP+Zer group exhibited a marked reduction in TRAP-stained osteoclast numbers, indicating that Zer suppresses osteoclastogenesis (Figure 1F,G). These findings suggest that Zer attenuated experimental periodontitis in male mice.

### 3.2. Zer Alleviated Local and Systemic Inflammatory Responses in Male Mice with Periodontitis

Given the central role of inflammation in bone resorption, we evaluated periodontal and systemic inflammatory markers. H&E staining in the CP group showed extensive inflammatory cell infiltration and structural disorganisation, which was markedly alleviated by Zer treatment (Figure 2A). Cytokine expression in gingival tissues was assessed by qRT-PCR. The mRNA levels of the pro-inflammatory cytokines *Il1b* and *Tnf* were elevated in the CP group relative to the Con group; the oral administration of Zer reversed this trend (Figure 2B,C). Consistently, serum ELISA revealed elevated circulating concentrations of IL-1β, IL-6, TNF-α and MPO in the periodontitis model group; these effects were attenuated by the Zer treatment (Figure 2D–G). Thus, Zer attenuated both local and systemic inflammation in male mice with periodontitis.

To assess potential biotoxicity, serum biomarkers, including AST and ALT as markers of liver function, together with BUN and Cr for renal function, were measured. Statistical analysis revealed no statistically significant variation among the three groups (Figure 2H–K), suggesting no overt hepatorenal toxicity under the tested conditions, consistent with previous studies [23,26].

### 3.3. In Silico Target Screening and Functional Annotation of Zer in Periodontitis

We next employed a network pharmacology strategy to investigate the potential mechanisms underlying the protective effect. The candidate targets of Zer were retrieved with the SwissTargetPrediction, GeneCards, and SEA databases. After standardising the gene symbols using the UniProt database and removing duplicates, 137 targets were retained. Concurrently, candidate genes associated with periodontitis were screened from five public databases: GeneCards (3501 targets, 1606 retained after filtering for a score ≥ 1), OMIM (2 targets), DisGeNET (682 targets), and CTD (12,264 targets). Following the removal of duplicates, a total of 2792 periodontitis-related target genes were compiled. Intersection analysis identified 72 overlapping targets between Zer and periodontitis (Figure 3A), which were designated as the candidate therapeutic targets for subsequent investigations (raw data provided in Supplementary Materials).

To prioritise the key nodes within these 72 candidates, a PPI network was built via STRING and visualised using Cytoscape (Figure 3B). The core targets included *NFKB1*, *MAPK14*, and *AKT1*, which were identified as the topologically central candidate targets of Zer in the context of periodontitis.

To decipher the biological relevance of these 72 candidates, GO and KEGG enrichment analyses were performed. KEGG enrichment analysis revealed 138 statistically significant pathway terms (*p* < 0.05), with the most relevant including apoptosis, lipid and atherosclerosis, and the IL-17 signalling pathway (Figure 3C). For further analysis, we focused on IL-17A, the key effector of the IL-17 signalling pathway [30]. This pathway was selected because it was robustly enriched in our KEGG data, covered two of our predicted targets (*NFKB1* and *MAPK14*), and has been repeatedly linked to periodontitis [31]. GO term analysis (Figure 3D) showed marked enrichment in 1419 biological processes (BP), 14 cellular components (CC), and 74 molecular functions (MF) (*p* < 0.05). The top-ranked BP terms involved “cellular response to chemical stress”, “response to toxic substance”, and “response to oxidative stress”. The CC terms included the endosome lumen, secretory granule lumen, and cytoplasmic vesicle lumen. The MF terms comprised RNA polymerase II-specific DNA-binding transcription factor binding, DNA-binding transcription factor binding, and nuclear receptor activity.

### 3.4. Network Pharmacology and Single-Cell Transcriptomics Suggest Distinct Cellular Localisation of Core Targets

To integrate the predicted targets into a pharmacological framework, a “Zer-target-pathway” interaction network was constructed by linking Zer, the 72 overlapping targets, and ten KEGG pathways critically involved in the inflammatory response (Figure 4A).

To anchor these computational predictions (*NFKB1*, *MAPK14*, *AKT1* and *IL17A*) within the periodontal microenvironment, we analysed the public scRNA-seq dataset GSE171213 from human periodontal tissues. Using canonical lineage markers (e.g., *MS4A6A* for macrophages, *TRAC* for T cells), all cells were systematically classified into ten major cell types (Figure 4B). Figure 4C displays the distribution of these populations in HC and PD samples. Feature plot analysis revealed distinct expression patterns for the core targets: *NFKB1*, *MAPK14*, and *AKT1* were predominantly expressed in macrophage clusters (Figure 4D–F), whereas *IL17A* was primarily localised to T cells (Figure 4G).

### 3.5. Molecular Docking and MD Simulations Predict Binding Modes of Zer to Core Targets

To characterise the binding modes, we docked Zer against its core predicted targets (NF-κB p50, p38 MAPK, AKT1, and IL-17A) using AutoDock 4.2. Zer inserted into the active pockets of NF-κB p50 (Figure 5A), p38 MAPK (Figure 5B), AKT1 (Figure 5C), and IL-17A (Figure 5D), establishing hydrogen bonds and hydrophobic contacts with crucial residues, with binding energies spanning −5.70 to −7.14 kcal·mol^−1^ (Table 2).

To assess the dynamic stability of these complexes, MD simulations extending to 100 ns were run with GROMACS 2023.5 using the Amber99SB-ILDN force field. RMSD trajectories (Figure 5E,I,M,Q) reached plateaus by approximately 20 ns for all complexes, with mean values of 1.14 nm (Zer-NF-κB p50), 0.22 nm (Zer-p38 MAPK), 0.23 nm (Zer-AKT1), and 0.33 nm (Zer-IL-17A), with fluctuations generally within ± 0.1 nm (Table 3); the Zer-NF-κB p50 complex plateaued as early as within 10 ns, albeit at a higher mean value compared to the others. The Zer-p38 MAPK complex showed a slight RMSD increase after 55 ns, stabilising at 0.3–0.4 nm for the remainder of the simulation. RMSF (Figure 5F,J,N,R), Rg (Figure 5G,K,O,S), and SASA (Figure 5H,L,P,T) analyses indicated that binding-pocket residues remained flexible, the complexes maintained compact globular conformations, and no major solvent exposure occurred. Collectively, these in silico data suggest stable binding of Zer to its predicted targets, providing a structural basis for our subsequent in vitro validation.

### 3.6. Zer Inhibited LPS-Induced Inflammatory Cytokine Expression and M1 Polarisation in RAW264.7 Macrophages

Based on the scRNA-seq findings implicating macrophages in Zer’s mechanism, we focused on this cell type for in vitro validation. The impact of Zer on LPS-driven inflammatory cytokine release was examined in RAW 264.7 cells. A CCK-8 assay was first carried out to evaluate its effect on cell viability. Zer did not significantly affect cell viability at 3 μM or 15 μM, whereas 30 μM significantly decreased viability. Accordingly, 3 μM (Z-L) and 15 μM (Z-H) were selected for later experiments (Figure 6A). ELISA revealed that following LPS challenge of RAW 264.7 cells, the addition of Z-L or Z-H attenuated the release of the pro-inflammatory cytokines IL-1β, IL-6, and TNF-α (Figure 6B–D).

To examine the role of Zer in macrophage polarisation, we analysed markers associated with M1 and M2 phenotypes. Zer treatment reversed the LPS-triggered increase in M1 markers (*Il1b*, *Tnf*, and *Nos2*; Figure 6E–G) and promoted a shift towards an M2-like phenotype. This was evidenced by increased expression of *Il10* and *Arg1* following Zer co-treatment, relative to the levels observed with LPS alone (Figure 6H,I). Furthermore, IF staining performed 24 h after treatment revealed that LPS significantly increased iNOS protein levels compared with the control group, which indicates M1 macrophage polarisation. This LPS-induced elevation of iNOS was suppressed by Zer (Figure 6J,K). It is notable that the measured factors are established markers for macrophage polarisation states: *Il1b*, *Il6*, *Tnf*, and *Nos2* for the pro-inflammatory M1 phenotype, and *Il10* and *Arg1* for the anti-inflammatory M2 phenotype [7,32].

### 3.7. Zer Suppressed IL-17A Expression and LPS-Driven Phosphorylation of NF-κB, p38 MAPK, and AKT1 in Macrophages

To further examine our computational predictions, we investigated the effects of Zer on key signalling pathways implicated by network pharmacology. IHC staining showed that Zer significantly reduced the elevated IL-17A expression in periodontal lesions of male mice (Figure 7A,B). We next examined the phosphorylation status of NF-κB p65, p38 MAPK, and AKT1 in macrophages by WB (Figure 7C). Upon LPS stimulation, both Z-L and Z-H downregulated LPS-induced phosphorylation of p65, p38 MAPK, and AKT1; at 30 or 60 min post-stimulation, the Z-H group presented more pronounced downregulation compared with the Zer-L group (Figure 7D–F). Together, these findings indicate that Zer suppresses LPS-induced phosphorylation of NF-κB, p38 MAPK and AKT1 in macrophages while reducing IL-17A expression in vivo, consistent with our network pharmacology predictions.

## 4. Discussion

To elucidate the mechanism of Zer in periodontitis, we combined in vitro and in vivo experiments with computational methods including network pharmacology, molecular docking and MD simulations. Public single-cell transcriptomic data were also analysed to assign candidate targets to specific cell types. Our results show that Zer attenuates alveolar bone resorption, local gingival inflammation, and systemic inflammatory markers in the serum of male mice with periodontitis. These findings are consistent with earlier reports of Zer’s anti-inflammatory activity in other inflammatory disorders, including arthritis [33] and colitis [34,35] where it modulates oxidative stress and cytokine cascades, and corroborate its tissue-reparative properties, which have been documented in ocular trauma [18]. By integrating single-cell transcriptomic profiling with computational target prediction, we therefore propose macrophage NF-κB, p38 MAPK, and AKT1 signalling as predicted target pathways that may mediate its immunomodulatory actions.

Computational prediction combined with single-cell transcriptomics predicted three macrophage-enriched targets—*NFKB1*, *MAPK14* and *AKT1*—and a T-cell-restricted target, *IL17A*. The macrophage-enriched expression of *NFKB1* and *MAPK14* is consistent with their known involvement in periodontal inflammation. NF-κB and p38 MAPK are well-established mediators in periodontitis [8,36], and their identification as hub targets aligns with previous network pharmacology studies of natural products in inflammatory conditions [37,38], supporting both the relevance of our screening strategy and the hypothesis that Zer acts on macrophage inflammatory signalling. AKT1, though also enriched in macrophages, has a more context-dependent role [39,40]; we therefore examined its phosphorylation in subsequent macrophage model. The T-cell-restricted localisation of *IL17A* suggests a potential involvement of T cells in the periodontal response, but this is not addressed by our current data. Molecular docking predicted favourable binding of Zer to these targets, with binding energies in the range previously associated with stable interactions. MD simulations further supported these predictions: the predicted complexes remained stable throughout the simulation period, with key contacts maintained. The slightly higher RMSD observed for Zer–NF-κB p50 likely reflects loop flexibility rather than destabilisation, consistent with stable Rg/SASA values, while the minor fluctuation for Zer–p38 MAPK did not affect the binding mode. These computational predictions suggest that Zer may interact stably with these targets, but direct experimental validation is required to determine how they contribute to the immunomodulatory effects of Zer. Among these targets, the *NFKB1* gene product, p105, is proteolytically processed to yield p50, the DNA-binding subunit of the canonical NF-κB transcription factor [41,42].

To determine whether the predicted targeting of *NFKB1* is associated with functional suppression of the NF-κB pathway, we measured p65 phosphorylation, as p50-p65 heterodimerisation is essential for transcriptional activation [43,44]. Following LPS stimulation of RAW 264.7 macrophages, Zer dose-dependently suppressed LPS-induced phosphorylation of NF-κB p65, p38 MAPK and AKT1. These effects were associated with reduced secretion of IL-1β, IL-6, and TNF-α; and a shift at the mRNA level from M1 markers (*Il1b*, *Tnf*, *Nos2*) towards M2 markers (*Il10*, *Arg1*). Co-suppression of these three nodes is consistent with the known activation of PI3K-AKT and canonical inflammatory pathways by LPS [45], and with previous reports that blocking these pathways is anti-inflammatory [46]. The decline in p65 phosphorylation is consistent with the network-predicted targeting of *NFKB1*. Since p50 requires heterodimerisation with p65 for transcriptional activity [41,42], binding of Zer to the p50 dimerisation interface or DNA-binding groove is predicted to sterically hinder this interaction (p65 is encoded by *RELA*), a prerequisite for transcriptionally active NF-κB complexes [47]. This may explain the observed reduction in p-p65 (Figure 7C). Nonetheless, we acknowledge that potential effects on upstream kinases (e.g., the IKK complex) or receptor-proximal signalling events cannot be excluded from the present data [6]. The concurrent reduction in AKT1 phosphorylation and the M2-skewed phenotype may appear counterintuitive, given the context-dependent roles of PI3K/AKT signalling in macrophages [39,40]. However, the net effect of AKT inhibition on polarisation is known to depend on isoform specificity and signal duration. In certain inflammatory contexts, AKT1 activation has been linked to pro-inflammatory responses, such as increased immune cell migration in experimental arthritis [46]. One possible explanation is that reduced AKT1 phosphorylation attenuates such pro-inflammatory signalling rather than directly driving M2 polarisation. Finally, while our in vitro experiments were performed exclusively in macrophages —which do not produce IL-17A—and do not directly address the IL-17 pathway or T-cell-derived IL-17A, the reduced gingival IL-17A observed in vivo remains a correlative observation. It raises the possibility that Zer may indirectly modulates T-cell-mediated immune responses, but this hypothesis requires direct experimental testing in appropriate T-cell or co-culture systems.

The in vivo reduction in TRAP-positive osteoclasts and gingival IL-17A suggests that Zer may also influence osteoclastogenesis and T-cell-associated responses indirectly. Specifically, the reduction in osteoclasts is consistent with the established roles of NF-κB and p38 MAPK in RANKL-induced osteoclast differentiation [48,49] and with the pro-osteoclastogenic effects of inflammatory cytokines [50]. Since macrophages are a major source of these cytokines in periodontitis, this effect is most likely mediated indirectly through Zer’s suppression of macrophage inflammatory signalling. However, this interpretation is limited by the absence of osteoclast differentiation assays or macrophage-specific interventions to directly confirm the indirect pathway. The decrease in IL-17A in vivo correlates with the IL-17 pathway enrichment predicted by network pharmacology. Given the known crosstalk between M1 macrophages and Th17 cells [51,52,53,54], this may reflect an indirect effect of Zer on T-cell responses, although it remains correlative. Nevertheless, the macrophage signalling data provide robust evidence for Zer’s anti-inflammatory effects on this cell type. The in vivo observations, including the reduction in IL-17A, may be compatible with this interpretation.

In this study, Zer was administered systemically and is therefore proposed as a systemic host-modulatory adjunct [8,15], not a locally delivered agent. Mechanical debridement of the subgingival biofilm remains the mainstay of periodontal therapy [9], but may not fully eliminate pathogens or resolve residual inflammation in all cases; adjunctive pharmacotherapy may therefore benefit selected patients [15]. Zer may serve this role by dampening inflammation and bone resorption. However, our post-induction protocol differs from the chronic clinical scenario in which patients typically present with longstanding disease; the optimal timing and duration of Zer treatment in humans therefore remain to be defined. In addition, local delivery (e.g., subgingival irrigation or controlled-release formulations) could reduce systemic exposure and improve target-site availability [55]. Whether systemic or local delivery is preferable remains to be tested. Future studies should compare these approaches and evaluate Zer as an adjunct to mechanical debridement.

Although Zer was effective in this murine model, these findings represent experimental efficacy only and do not establish clinical efficacy in human periodontitis; several further obstacles remain before clinical translation can be considered. Zer is poorly water-soluble and has a relatively short half-life (T_1_/_2_ = 5.93 h) [56], factors that may limit its oral bioavailability and exposure in human tissues. We did not measure Zer concentrations in gingival tissue or serum, so whether the in vitro concentrations (3–15 μM) are achievable in vivo remains to be determined. Its efficacy relative to existing host-modulation therapies for periodontitis—anti-cytokine biologics and emerging small-molecule inhibitors [55]—has not been tested head-to-head. Safety beyond our 2-week assessment also remains uncharacterised. Addressing these gaps in pharmacokinetics, comparative efficacy, and long-term safety will be necessary before clinical translation can be considered.

Taken together, by integrating in vivo phenotyping, in vitro mechanistic assays, and computational predictions with single-cell transcriptomic localisation, this study suggests macrophage NF-κB, p38 MAPK and AKT1 signalling as candidate mediators of Zer’s immunomodulatory actions in periodontitis. Several limitations should also be acknowledged. A notable limitation is that our findings are restricted to male mice; whether they extend to females remains to be determined and warrants further investigation. The single-cell data were derived from a public repository rather than from our own experiments, and the mechanistic limitations of our in vitro system are discussed above. Additionally, while we observed reduced AKT1 phosphorylation upon Zer treatment, our current data do not establish a causal link between this event and the M2-polarised phenotype. Functional validation using selective AKT inhibitors or isoform-specific knockdown would be required to definitively dissect the contribution of AKT1 in this context. Furthermore, the predicted molecular interactions need direct biophysical validation, for example by surface plasmon resonance (SPR) or cellular thermal shift assay (CETSA). Future studies using macrophage-specific conditional knockout or IL-17 neutralisation will be needed to clarify target dependency and the contribution of the IL-17/T-cell axis. Such experiments will be essential for validating the mechanism of Zer in periodontitis. Moreover, the limitations outlined above—including the use of male mice only, reliance on public single-cell data, absence of osteoclast/T-cell differentiation experiments, and lack of direct biophysical target validation—collectively constrain the translational interpretation of our findings. Therefore, the present results should be regarded as hypothesis-generating preclinical evidence to guide future research, rather than a basis for clinical recommendations.

## 5. Conclusions

In summary, by integrating computational prediction, public single-cell transcriptomic localisation, in vivo phenotyping, and in vitro mechanistic assays, this study suggests that macrophage NF-κB (p50/p65) signalling, p38 MAPK signalling and reduced AKT1 phosphorylation are signalling changes observed alongside Zer’s immunomodulatory effects in periodontitis. The accompanying reduction in gingival IL-17A further suggests a potential indirect effect on T cell responses. Collectively, these findings suggest that macrophage inflammatory signalling may be a potential immunomodulatory target of Zer, and they provide a rationale for further mechanistic studies in periodontitis. However, we acknowledge that the binding interactions between Zer and the candidate target proteins (NF-κB p50, p38 MAPK, AKT1) are based on computational docking and molecular dynamics simulations, and further biophysical validation (e.g., SPR, CETSA) is required to confirm direct target engagement.

## Figures and Tables

**Figure 1:**
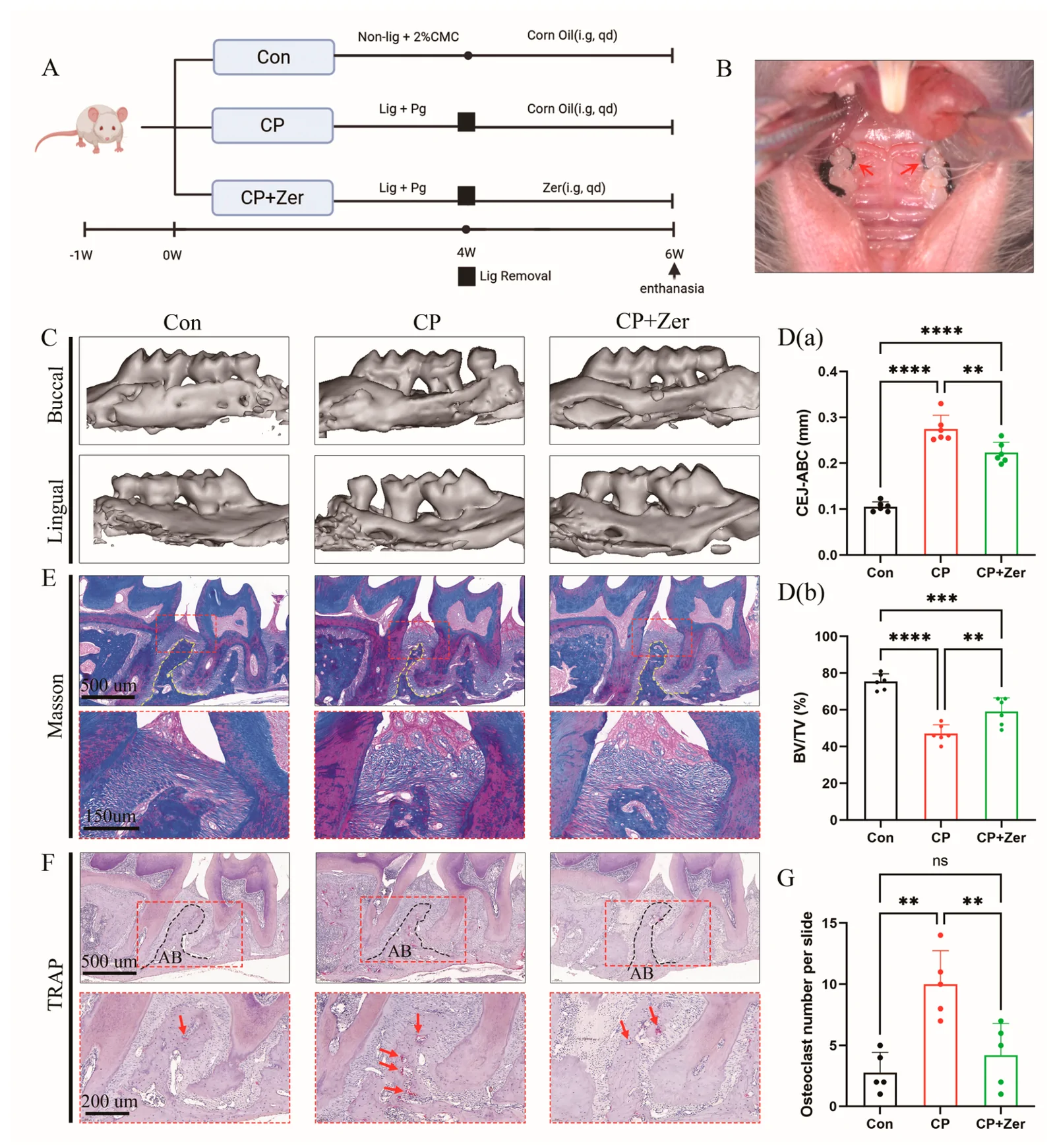
Zerumbone (Zer) attenuated alveolar bone resorption in male mouse models of periodontitis. (**A**) Male mouse grouping and treatment schedule. (**B**) Periodontitis was induced by placing bilateral silk ligatures within the maxillary second molar sulcus. The red arrow indicates the placement of the 5-0 silk suture used for the ligature. (**C**) Representative three-dimensional micro-CT images of the buccal and palatal views of the maxilla in the 3 groups of male mice, n = 6 per group. (**D**(**a**),**D**(**b**)) Alveolar bone resorption was quantified by measuring the CEJ-ABC distance and calculating the BV/TV ratio at the maxillary second molar, n = 6 per group. (**E**,**F**) Images of Masson’s trichrome and TRAP staining in male mouse periodontium (n = 5 per group). Red arrows point to osteoclasts. Scale bars: 500 µm, 200 µm, and 150 µm. (**G**) Quantitative assessment of the number of osteoclasts per section among the 3 groups, n = 5 per group. All n values represent biological replicates. Statistical significance was defined as ** *p* < 0.01, *** *p* < 0.001, **** *p* < 0.0001, ns, not significant. Abbreviations: AB, alveolar bone; BV/TV, bone volume/total volume; CEJ-ABC, distance from the cementoenamel junction to the alveolar bone crest; CMC, carboxymethyl cellulose; Con, control group; CP, periodontitis group; CP+Zer, periodontitis group treated with Zer; i.g, intragastrically; Lig, ligature; *Pg*, *Porphyromonas gingivalis*; qd, once daily; TRAP, tartrate-resistant acid phosphatase; Zer, zerumbone.

**Figure 2:**
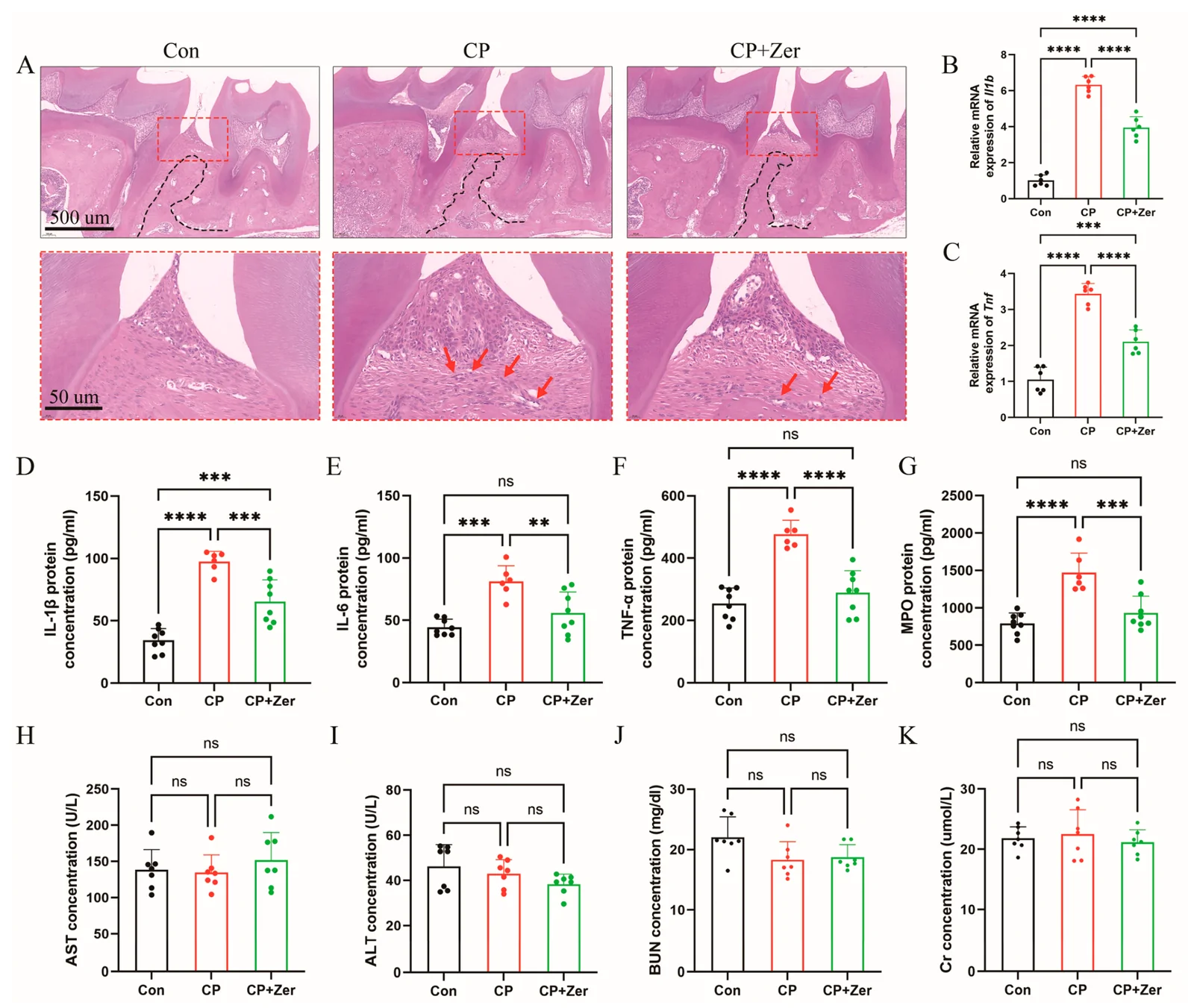
Zerumbone (Zer) reduced local and systemic inflammatory responses in male mice with periodontitis. (**A**) Representative H&E-stained cross-sections of maxillae from the three experimental groups (n = 6 per group). Red arrows point to inflammatory cells. Scale bars: 500 µm and 50 µm. (**B**,**C**) Expression of *Il1b* and *Tnf* mRNAs in gingival tissues, shown as relative mRNA levels. n = 6 per group. (**D**–**G**) Effects of Zer on the serum levels of IL-1β, IL-6, TNF-α and MPO in male mice. n = 6–8 per group (Con: 8; CP: 6; CP+Zer: 8). (**H**–**K**) Relative serum levels of liver and kidney injury biomarkers (AST, ALT, BUN, and Cr) in male mice. n = 7 per group. All n values represent biological replicates. For group comparisons, ** *p* < 0.01, *** *p* < 0.001, and **** *p* < 0.0001 were considered significant; ns, not significant. Abbreviations: Con, control group; CP, periodontitis group; CP+Zer, periodontitis group treated with Zer; Zer, zerumbone.

**Figure 3:**
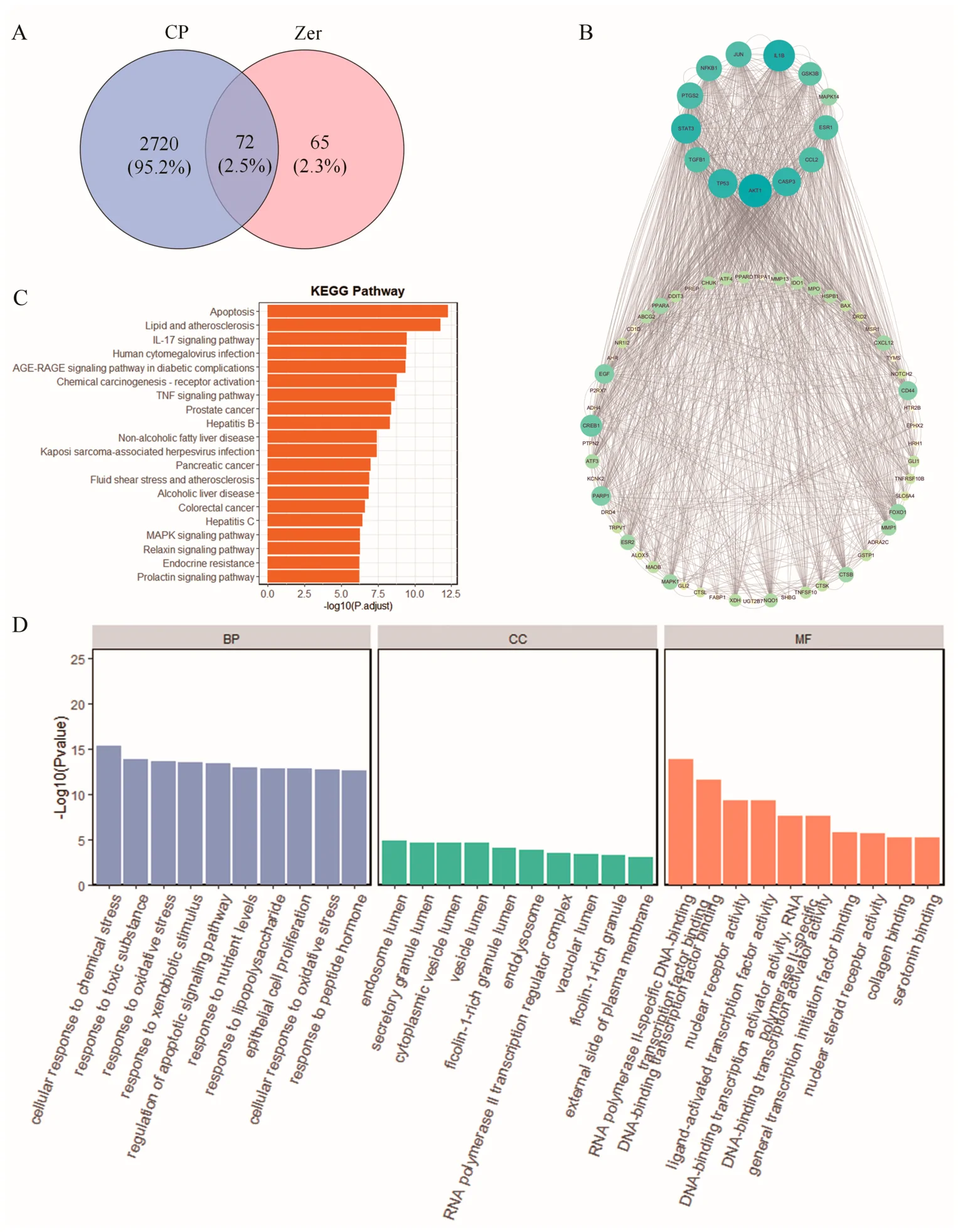
Identification, PPI network, and functional enrichment of overlapping targets between zerumbone (Zer) and periodontitis (CP). (**A**) Venn diagram of overlapping targets between Zer’s putative targets and periodontitis-related genes (Zer-CP-related genes). (**B**) PPI network of periodontitis-related genes targeted by Zer. (**C**) KEGG enrichment analysis of Zer’s anti-periodontitis mechanisms. The plot displays pathway terms on the Y-axis against their enrichment scores on the X-axis. (**D**) The top 10 significant GO terms (BP/CC/MF) ranked according to *p*-value. The Y-axis displays the enrichment counts of target genes, while the X-axis corresponds to their respective GO categories. Abbreviations: BP, biological process; CC, cellular component; CP, periodontitis; GO, Gene Ontology; KEGG, Kyoto Encyclopedia of Genes and Genomes; MF, molecular function; PPI, protein–protein interaction; Zer, zerumbone.

**Figure 4:**
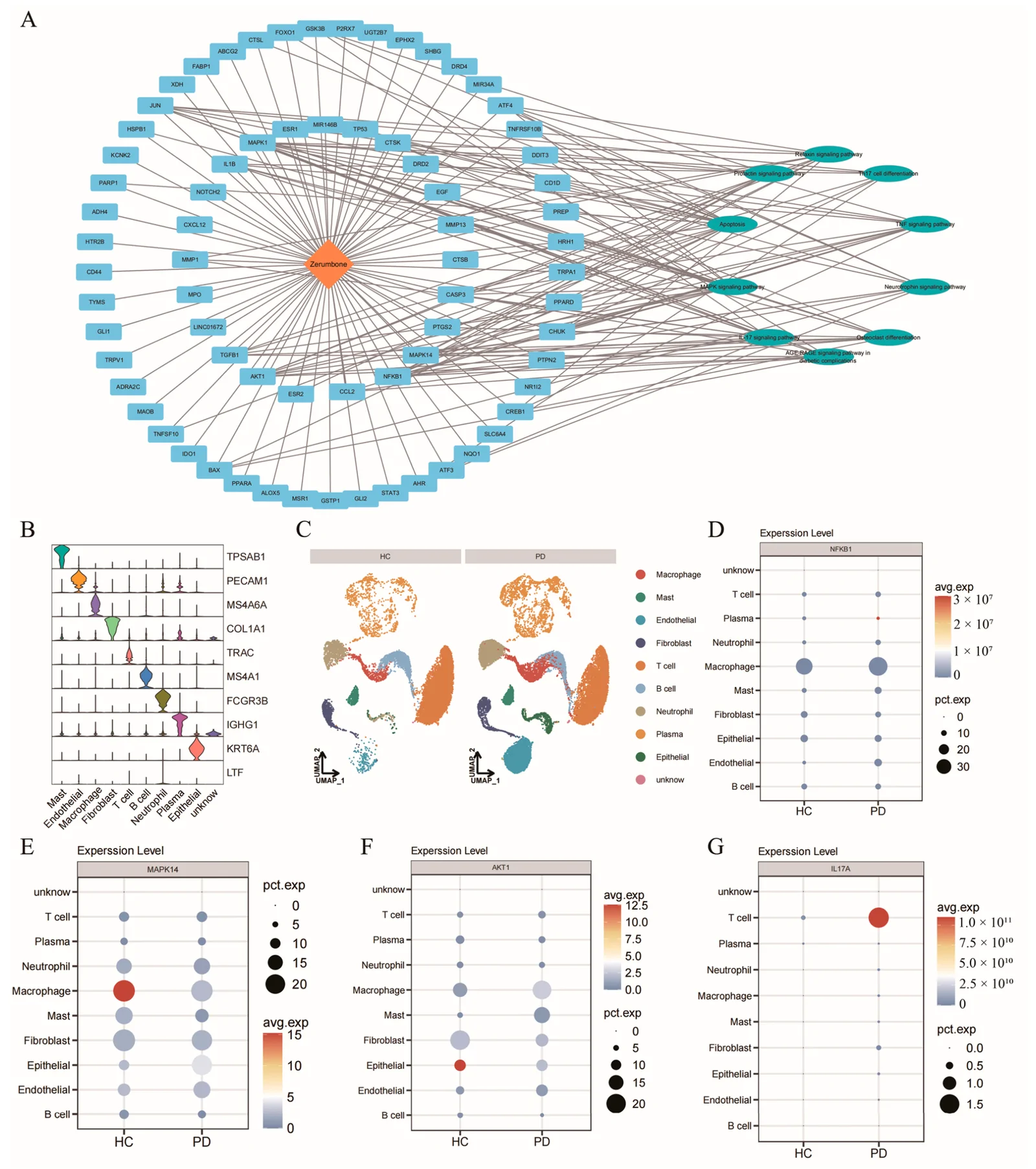
Zer-target-pathway network and scRNA-seq analysis of hub genes in periodontitis. (**A**) Target-pathway network of Zer in periodontitis therapy. Zer is depicted in red, pivotal targets are highlighted in green, and crucial pathways are denoted in blue. (**B**) Stacked violin plots depicting the expression profiles of cell type-specific markers in periodontal tissues. (**C**) UMAP visualisation of cell-type classification based on scRNA-seq data from the GSE171213 dataset. (**D**–**G**) Dot plots visualising the expression of *NFKB1* (**D**), *MAPK14* (**E**), *AKT1* (**F**) and *IL17A* (**G**) across cell clusters in the healthy control (HC) versus periodontally diseased (PD) groups. The dot size represents the relative abundance of cells showing marked gene expression in specific cellular subtypes, and the color intensity reflects the mean expression level across the expression-positive cells. Abbreviations: HC, healthy control; PD, periodontally diseased group; scRNA-seq, single-cell RNA sequencing; UMAP, uniform manifold approximation and projection; Zer, zerumbone.

**Figure 5:**
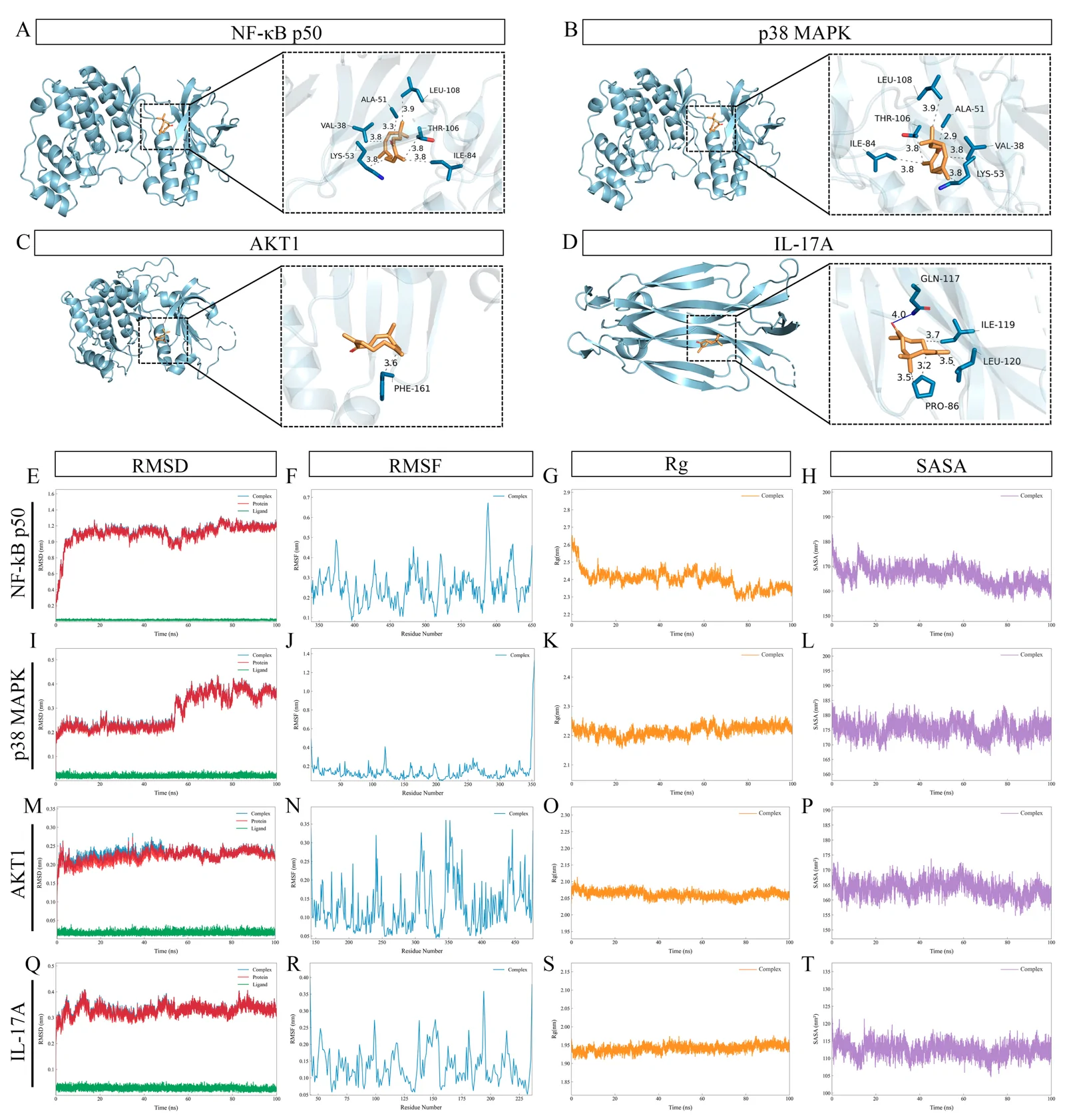
Molecular docking and MD simulation of Zer with NF-κB p50, p38 MAPK, AKT1, and IL-17A. (**A**–**D**) Molecular docking results of Zer with NF-κB p50, p38 MAPK, AKT1, and IL-17A. Target proteins are depicted in light blue, small-molecule ligands in yellow, oxygen atoms in red, interacting amino acid residues in blue, hydrogen bonds as solid blue lines, and hydrophobic interactions as black dashed lines. (**E**–**T**) MD simulation for each complex: RMSD, RMSF, Rg, and SASA plots for Zer- NF-κB p50 (**E**–**H**), Zer-p38 MAPK (**I**–**L**), Zer-AKT1 (**M**–**P**), and Zer-IL-17A (**Q**–**T**), assessing structural stability, residue flexibility, compactness, and solvent accessibility, respectively. Abbreviations: MD, molecular dynamics; Rg, radius of gyration; RMSD, root-mean-square deviation; RMSF, root-mean-square fluctuation; SASA, solvent-accessible surface area; Zer, zerumbone.

**Figure 6:**
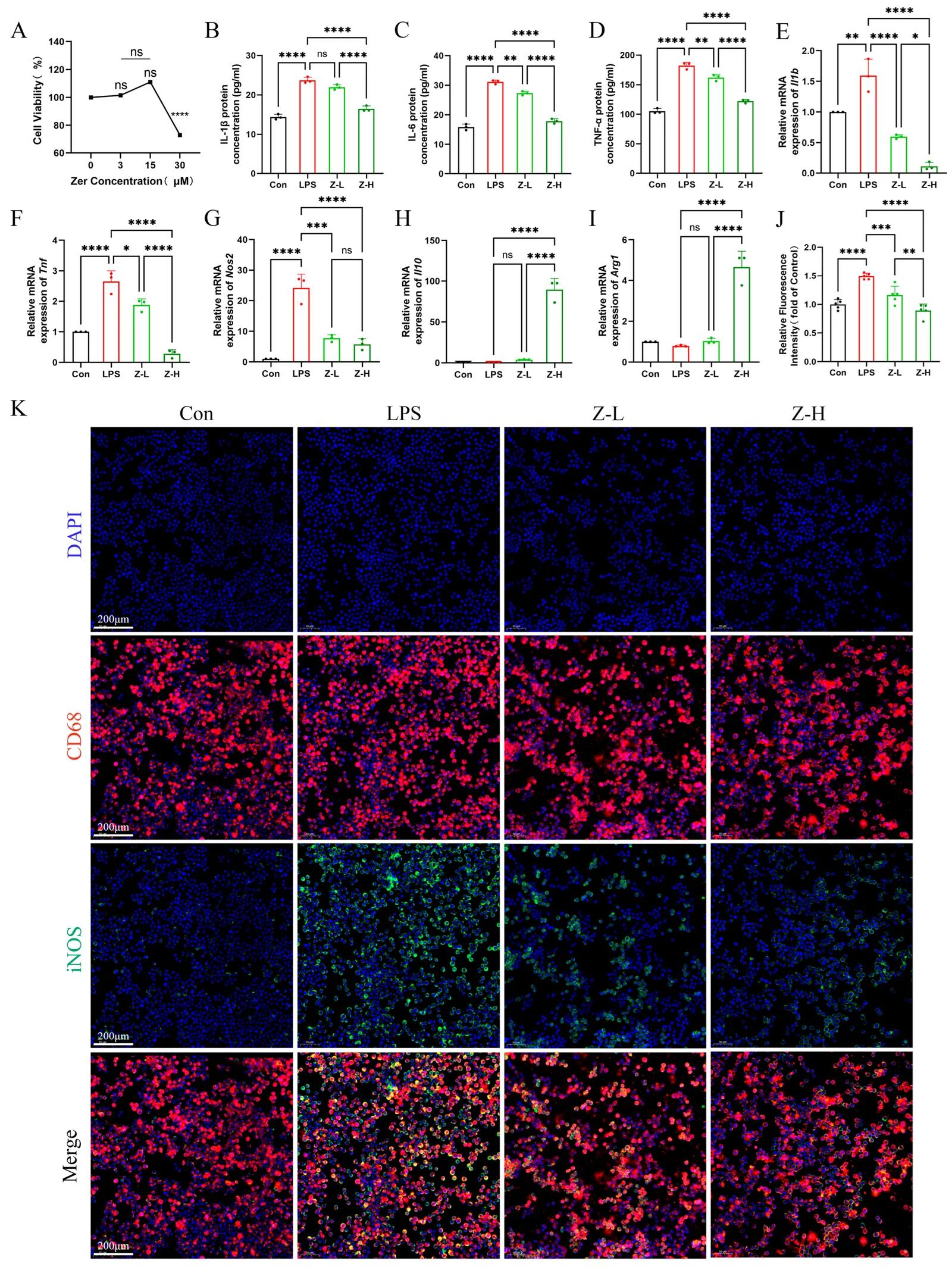
Zer attenuates LPS-induced M1 polarisation and inflammatory responses in macrophages in vitro. (**A**) Cell viability was evaluated using CCK-8 assay after exposure to 0, 3, 15, and 30 µM Zer (n = 3 per group). (**B**–**D**) Supernatant concentrations of IL-1β, IL-6 and TNF-α were determined via ELISA (n = 3 per group). (**E**–**I**) Relative mRNA levels of the indicated genes (*Il1b*, *Tnf*, *Nos2*, *Il10*, and *Arg1*; n = 3 per group). (**J**,**K**) Representative immunofluorescence images of iNOS (green) and the macrophage marker CD68 (red) in the four experimental groups, with semi-quantitative analysis performed only for iNOS; nuclei were visualised with DAPI (blue) (n = 5 per group). All n values represent biological replicates; technical replicates, where applicable, were averaged before analysis. Data are shown as mean ± SD. Significance levels: * *p* < 0.05, ** *p* < 0.01, *** *p* < 0.001, **** *p* < 0.0001; ns, not significant. Abbreviations: Arg1, arginase 1; CCK-8, cell counting kit-8; Con, control group; DAPI, 4′,6-diamidino-2-phenylindole; IL-10, interleukin 10; iNOS, inducible nitric oxide synthase; LPS, lipopolysaccharide-stimulated group; SD, standard deviation; Zer, zerumbone; Z-H, LPS + high-dose Zer (15 µM) group; Z-L, LPS + low-dose Zer (3 µM) group.

**Figure 7:**
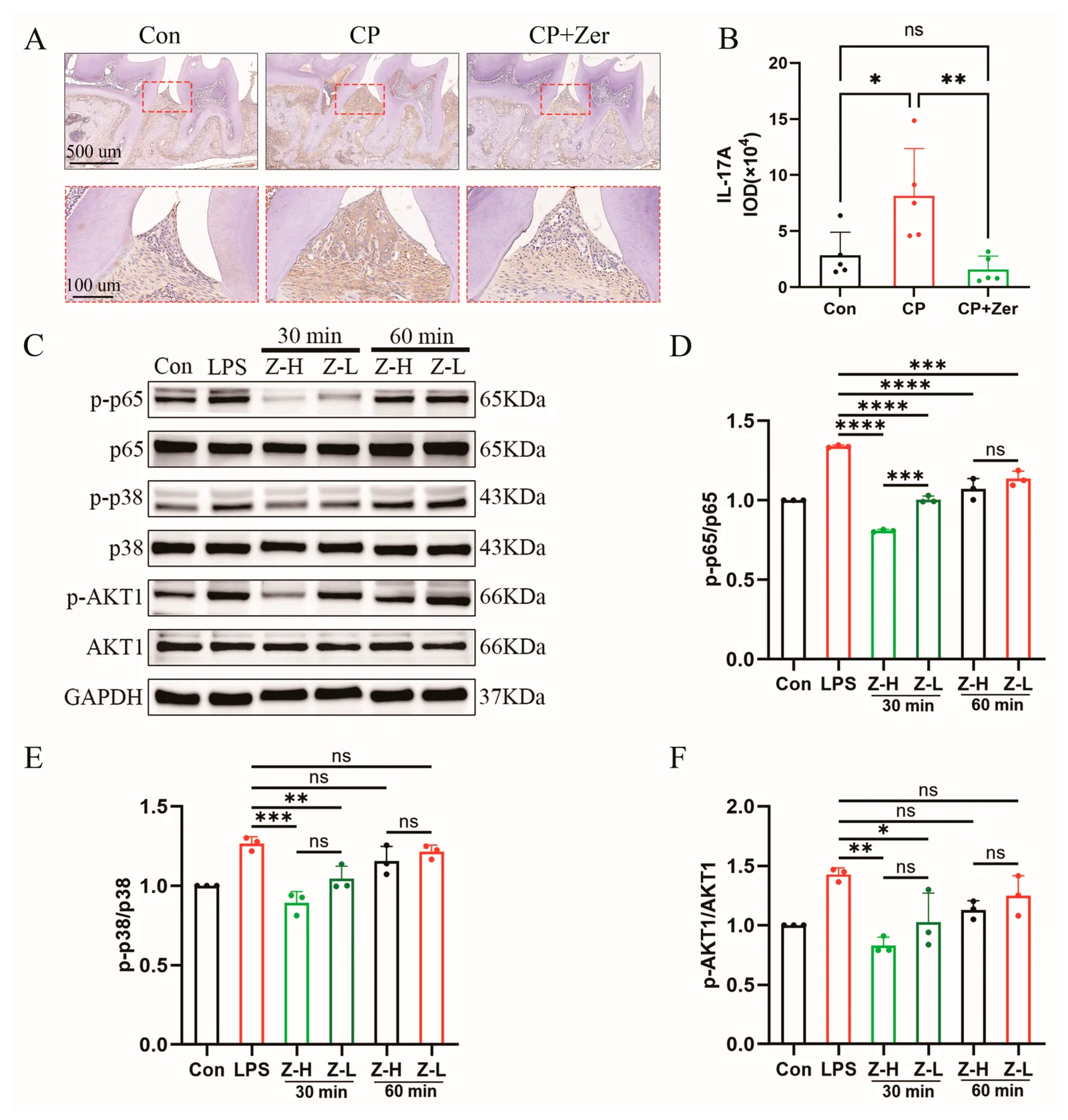
Validation of the network pharmacology results. (**A**) Representative IHC staining for IL-17A in periodontal tissues (n = 5 per group). Scale bars: 500 µm and 100 µm. (**B**) IL-17A expression within the stained regions was quantified using ImageJ. Data are shown as IOD (×10^4^). (**C**) The expression levels of NF-κB p65, p38 MAPK, AKT1 and their phosphorylated proteins were measured by Western blotting. Quantification of phosphorylated protein levels normalised to total protein for (**D**) p-p65, (**E**) p-p38 and (**F**) p-AKT1 levels; the experiments were repeated 3 times. All n values represent biological replicates; technical replicates, where applicable, were averaged before analysis. Data are shown as mean ± SD. Statistical significance: * *p* < 0.05, ** *p* < 0.01, *** *p* < 0.001, **** *p* < 0.0001; ns, not significant. Abbreviations: Con, control group; CP, periodontitis group; CP+Zer, periodontitis group treated with Zer; IHC, immunohistochemistry; IOD, integrated optical density; LPS, lipopolysaccharide-stimulated group; Zer, zerumbone; Z-H, LPS + high-dose Zer (15 µM) group; Z-L, LPS + low-dose Zer (3 µM) group.

**Table 1:** Primer sequences used in this study.

Genes	Forward (5′-3′)	Reverse (5′-3′)
*Il1b*	GAAATGCCACCTTTTGACAGTG	TGGATGCTCTCATCAGGACAG
*Tnf*	GCCGATGGGTTGTACCTTGT	TCTTGACGGCAGAGAGGAGG
*Nos2*	GGAGTGACGGCAAACATGACT	TCGATGCACAACTGGGTGAAC
*Il10*	CTTACTGACTGGCATGAGGATCA	GCAGCTCTAGGAGCATGTGG
*Arg1*	CAGCAGAGGAGGTGAAGAGTA	TAGTCAGTCCCTGGCTTATGG
*Gapdh*	TGAGGTGACCGCATCTTCTTG	TGGTAACCAGGCGTCCGATA

**Table 2:** Binding energies of Zer with potential target proteins.

Protein Target	Binding Energy (kcal·mol^−1^)
NF-κB p50	−5.70
p38 MAPK	−6.32
AKT1	−7.01
IL-17A	−7.14

Note: Zer, zerumbone.

**Table 3:** Summary of RMSD values for each complex during the equilibrated phase of the 100 ns molecular dynamics simulations.

Complex	Equilibration Time (ns)	Average RMSD (nm)	SD (nm)
Zer-NF-κB p50	20–100	1.14	0.07
Zer-p38 MAPK	20–100	0.22	0.01
Zer-AKT1	20–100	0.23	0.01
Zer-IL-17A	20–100	0.33	0.02

Note: RMSD, root mean square deviation; Zer, zerumbone; SD, standard deviation.

## Data Availability

The data supporting this study are accessible on Zenodo at https://doi.org/10.5281/zenodo.20026394 (accessed on 23 September 2026).

## Supplementary Materials

The following supporting information can be downloaded at https://www.mdpi.com/article/10.3390/cimb48100985/s1.

## Abbreviations

The following abbreviations are used in this manuscript:

3D	three-dimensional
ABC	alveolar bone crest
ALT	alanine aminotransferase
AST	aspartate aminotransferase
BP	biological process
BUN	blood urea nitrogen
BV	bone volume
CC	cellular component
CEJ	cementoenamel junction
CETSA	cellular thermal shift assay
CFU	colony-forming unit
CMC	carboxymethyl cellulose
Cr	creatinine
DMSO	dimethyl sulfoxide
ELISA	enzyme-linked immunosorbent assay
FDR	false discovery rate
GEO	Gene Expression Omnibus
GO	Gene Ontology
H&E	haematoxylin and eosin
HPLC	high-performance liquid chromatography
IF	immunofluorescence
IHC	immunohistochemistry
IOD	integrated optical density
KEGG	Kyoto Encyclopedia of Genes and Genomes
LPS	lipopolysaccharide
MD	molecular dynamics
MF	molecular function
micro-CT	micro-computed tomography
MPO	myeloperoxidase
OD	optical density
PBS	phosphate-buffered saline
PDB	Protein Data Bank
*Pg*	*Porphyromonas gingivalis*
PPI	protein–protein interaction
qRT-PCR	quantitative real-time PCR
Rg	radius of gyration
RMSD	root mean square deviation
RMSF	root mean square fluctuation
SASA	solvent-accessible surface area
scRNA-seq	single-cell RNA sequencing
SD	standard deviation
SPR	surface plasmon resonance
TRAP	tartrate-resistant acid phosphatase
TV	tissue volume
WB	Western blot
Zer	zerumbone
