# Supplementary material for: Integrated Identification of Macrophage NF-κB, p38 MAPK and AKT1 Signalling as Candidate Targets of Zerumbone in Experimental Periodontitis
Source: Curr Issues Mol Biol. 2026 Sep 24;48(10):985. doi: 10.3390/cimb48100985 (PMC13703733; doi:10.3390/cimb48100985)
Supplement: Supplementary file 1 [file cimb-48-00985-s001.zip › Supplementary_File_S1_Zerumbone_COA.pdf]

乐美天医药/德思特生物

Certificate of Analysis

Zerumbone

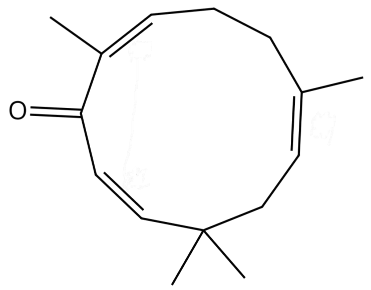

CAS Number: 471-05-6
Batch No.: DST230328-145
M. F.: $C_{15}H_{22}O$
M. W.: 218.34

Test Item	Specifications	Results
Appearance	White powder	Conforms
Purity (HPLC)	≥98.0%	99.77%
NMR	Comply with the structure	Conforms

Test Method	Column:Agilent Eclipse Plus C18 4.6*250 mm,5μm; Column temperature: 35℃; Detection Mode:UV248 nm; Flow Rate:1.0ml/min; Sample dissolution:Methanol;Mobile Phase:A-Acetonitrile ,B-0.1% Phosphoric acid in water;Gradient elution:A,60%-70%,20min,70%-90%,5min.
Storage	Keep Out of light, dry and stored at 2-8℃.
Hint	If you encounter quality problems, please contact us within 15 days after receiving the products and thank you for your purchase.
Note	The product is only used for scientific research experiments and should not be used for human injection, food or other purposes. This solution must be freshly prepared before using to avoid degradation of the purity of the product and affect the experimental results.

QC: chaonan zhong

QA: Chong chen

SpecialSeal: 质检章

成都德思特生物技术有限公司

Tel：028-85003950 Fax：028-85589956
http://www.lemeitian.cn Add：No. 128, West Section of Haike Road, Wenjiang District, Chengdu, Sichuan, China

Note: The seal translates as “ Quality Inspection Seal of Chengdu Desite Biotechnology Co., Ltd. ”

乐美天医药/德思特生物

Appendix 1：HPLC

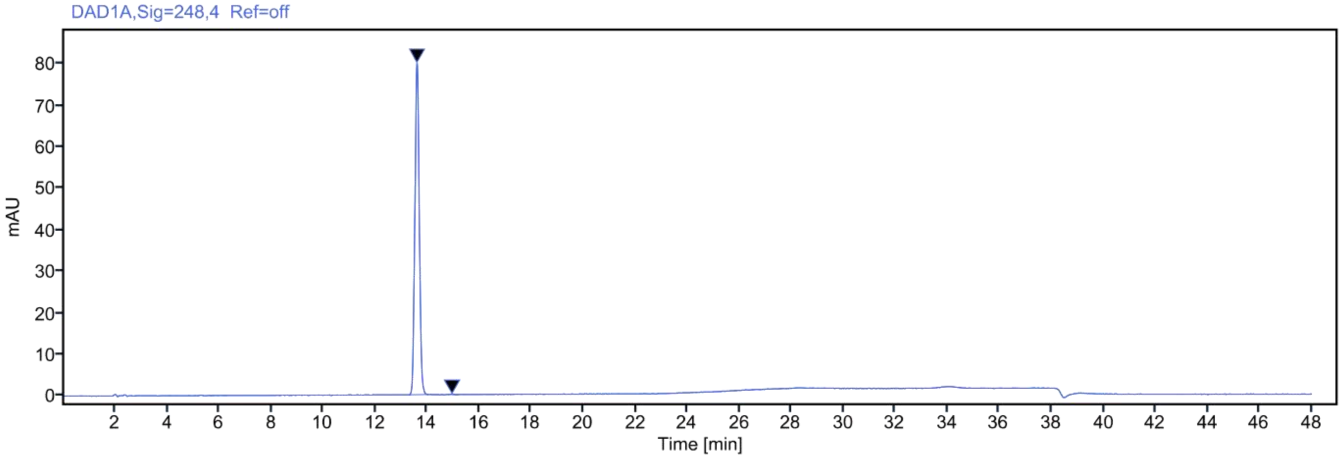

Signal: DAD1A,Sig=248,4 Ref=off

RT [min]	Type	Width [min]	Area	Height	Area%	Name
13.609	BB	0.75	944.49	79.88	99.77	-
14.955	MM m	0.40	2.16	0.18	0.23	
		**Sum**	**946.64**			

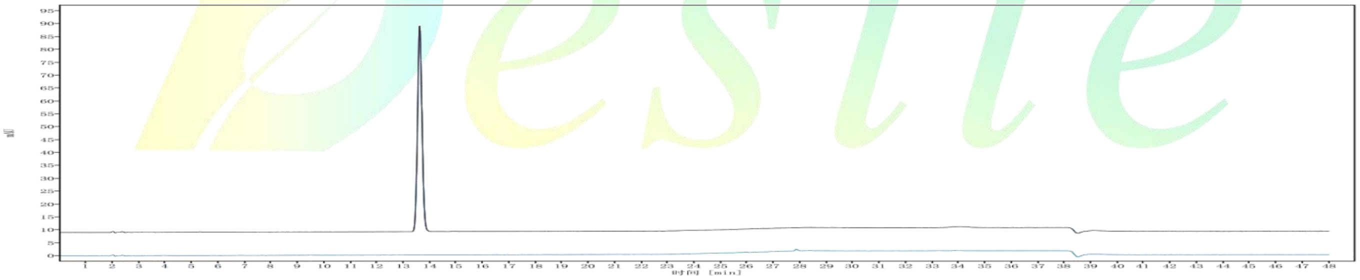

Tel：028-85003950 Fax：028-85589956
http://www.lemeitian.cn Add：No. 128, West Section of Haike Road, Wenjiang District, Chengdu, Sichuan, China

Appendix 2：1H-NMR

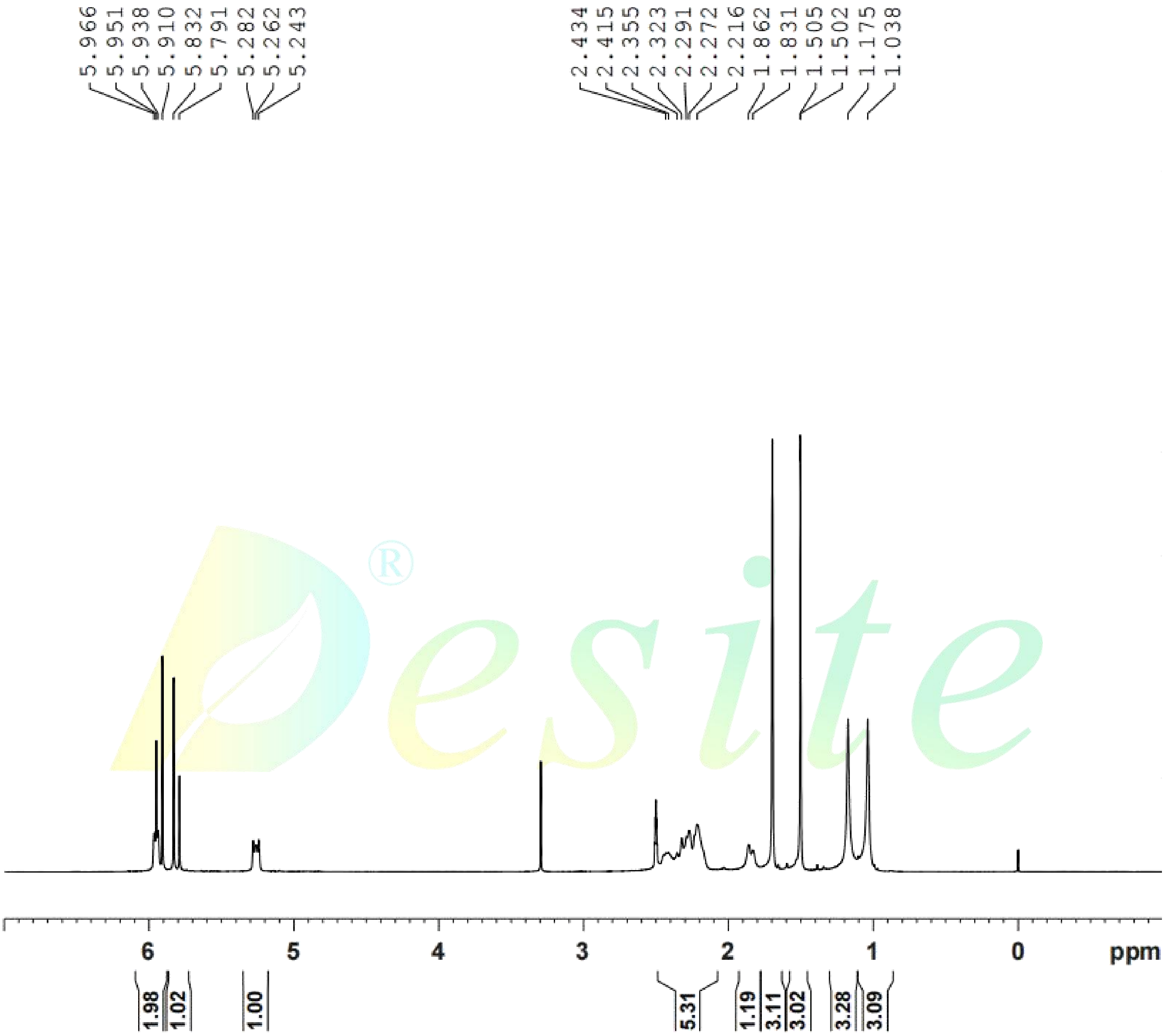

Tel：028-85003950 Fax：028-85589956
http://www.lemeitian.cn Add：No. 128, West Section of Haike Road, Wenjiang District, Chengdu, Sichuan, China
