# Supplementary material for: Integrated Identification of Macrophage NF-κB, p38 MAPK and AKT1 Signalling as Candidate Targets of Zerumbone in Experimental Periodontitis
Source: Curr Issues Mol Biol. 2026 Sep 24;48(10):985. doi: 10.3390/cimb48100985 (PMC13703733; doi:10.3390/cimb48100985)
Supplement: Supplementary file 1 [file cimb-48-00985-s001.zip › Supplementary_File_S2_Web_Links_and_URLs.pdf]

Web links and URLs:

1. The SwissTargetPrediction database (for zerumbone targets). http://swisstargetprediction.ch/. Accessed 21 December 2023.

2. The GeneCards database (for zerumbone targets). https://www.genecards.org/. Accessed 22 December 2023.

3. The SEA database (for zerumbone targets). https://sea.bkslab.org/. Accessed 11 August 2024.

4. The GeneCards database (for periodontitis targets). https://www.genecards.org/. Accessed 11 August 2024.

5. The OMIM database (for periodontitis targets). https://www.omim.org/. Accessed 4 August 2024.

6. The DisgeNET database (for periodontitis targets). https://www.disgenet.org/. Accessed 21 December 2023.

7. The CTD database (for periodontitis targets). https://ctdbase.org/. Accessed 11 August 2024.

8. The Venny 2.1 Online Website. (https://bioinfogp.cnb.csic.es/tools/venny/). Accessed 11 August 2024.

9. The STRING database. (https://cn.string-db.org/). Accessed 19 August 2024.

10. The RCSB Protein Data Bank database. (https://www.rcsb.org). Accessed 18 March 2026.
